# Exploring how healthcare teams balance the neurodynamics of autonomous and collaborative behaviors: a proof of concept

**DOI:** 10.3389/fnhum.2022.932468

**Published:** 2022-07-28

**Authors:** Ronald Stevens, Trysha L. Galloway

**Affiliations:** ^1^UCLA School of Medicine, Brain Research Institute, Los Angeles, CA, United States; ^2^The Learning Chameleon, Inc., Culver City, CA, United States

**Keywords:** teamwork, taskwork, EEG, hyperscanning, uncertainty, representation design, team neurodynamics, information theory

## Abstract

Team members co-regulate their activities and move together at the collective level of behavior while coordinating their actions toward shared goals. In parallel with team processes, team members need to resolve uncertainties arising from the changing task and environment. In this exploratory study we have measured the differential neurodynamics of seven two-person healthcare teams across time and brain regions during autonomous (taskwork) and collaborative (teamwork) segments of simulation training. The questions posed were: (1) whether these abstract and mostly integrated constructs could be separated neurodynamically; and, (2) what could be learned about taskwork and teamwork by trying to do so? The taskwork and teamwork frameworks used were Neurodynamic Information (*NI*), an electroencephalography (EEG) derived measure shown to be a neurodynamic proxy for the pauses and hesitations associated with individual uncertainty, and inter-brain EEG coherence (*IBC*) which is a required component of social interactions. No interdependency was observed between NI and IBC, and second-by-second dynamic comparisons suggested mutual exclusivity. These studies show that proxies for fundamental properties of teamwork and taskwork can be separated neurodynamically during team performances of ecologically valid tasks. The persistent expression of *NI* and *IBC* were not simultaneous suggesting that it may be difficult for team members to maintain inter-brain coherence while simultaneously reducing their individual uncertainties. Lastly, these separate dynamics occur over time frames of 15–30 s providing time for real-time detection and mitigation of individual and collaborative complications during training or live patient encounters.

## Introduction

Teams are a social response to recurring and required tasks that are too difficult for one person to accomplish. The dual complexities of teams and tasks result in evolutionary systems, with teams varying their behaviors in response to external perturbations and changing task requirements while also using their behaviors to constrain and shape the task (Ashby, 1956). As expected from the changing complexities of the task and the environment, teams are different from the natural flow of most human activities by being constrained by time, resources, and ability. In these dynamic decision-making moments, the changing individual and collaborative elements contributing to the success or failure of teams are difficult to identify, challenging our ability to predict future dynamics either by humans or machines (Stevens and Galloway, 2021a).

Historically the dynamics of teams have been observed through many lenses. These include intentions from the perspective of agents (Cohen and Levesque, 1990) or humans (Knoblich and Sebanz, 2008), shared plans and planning (Grosz and Kraus, 1996), joint actions (Sebanz et al., 2006) shared cognition (Gorman and Cooke, 2011), team mental models (Mohammed et al., 2010), team coordination (Gorman et al., 2010), and macrocognition (Klein et al., 2003). What these ideas share is an increased understanding of how well individuals can recognize and act appropriately on the intentions of others. While intention provides a social cognitive background for the shared actions of a team, the interdependencies of the joint actions provide the organizational structure within; and with that, teams perform joint tasks and realize common goals (Wageman, 2001; Kozlowski and Bell, 2020).

### Team functioning

When people collaborate on a task at least two simultaneous processes are thought to occur, taskwork and teamwork (Salas et al., 2004; Driskell et al., 2018). The taskwork of individuals is where their expertise and experience are used to develop information relevant to the team goals and convey it efficiently and effectively to others (Paris et al., 2000). The skills of taskwork are defined by the task domain, i.e., being a sonar operator or surgeon. Task skills are easier to define, observe, describe, and assess than team skills and can be measured using standard psychometric techniques (Von Davier and Halpin, 2013).

The skills of teamwork include those that help establish and support effective communication, problem-solving, management of resources, and managing conflict (Salas et al., 2007). These skills are usually taught by working together on-the-job, or through simulation-based training and are measured by expert observations (Baker et al., 2011) and the use of vetted rubrics (Jones et al., 2011); this is likely to change.

While our understanding of individual skill development is improving, our understanding of how to develop team skills often results in unanswered questions (Stevens et al., 2017): Are teams more (or less) than the sum of their parts? Can teamwork be separated from taskwork? What distinguishes the temporal and spatial dynamics of teamwork and taskwork, and are the boundaries between them discrete or continuous? Are the dynamics of these constructs independent, interdependent, or mutually exclusive at time scales (i.e., the hierarchical depth of cognition) that would have immediate relevance for planning and training? Answering such questions would have implications for: training (Fisher, 2014), assembling and enabling task vs. collaboration enhanced robots (Kamika, 2019) and for training artificial intelligence to forecast possible team outcomes based on their neurodynamics (Stevens and Galloway, 2021a).

The expanded repertoire and analytic capabilities of physiologic sensors are shifting the research lens once again, providing increasingly rich data and quantitative tools for describing the brain during increasingly complex situations (Kazi et al., 2021). This re-focusing of teamwork research emphasizes a shift toward more implicit (automatic, fast, subconscious) interactions (as opposed to observations) of team members during dynamic social interactions in ecologically valid, uncontrolled, and prolonged real-world tasks (Wiese et al., 2018; Abubshait et al., 2021). These capabilities and shifts toward ecologically valid settings are also causing researchers to re-think experimental designs and analyses, shifting toward representative design as a principled basis for ecological generalizability, taking complex phenomena and deconstructing them into manageable components (Nastase et al., 2020; De Sanctis et al., 2021; Gramann et al., 2021), where theoretical assumptions are relaxed at the stage of experimental design and data collection, and later imposed during different stages of analysis.

Looking forward, we are now at a point where the practical insertion of these technologies into improving teamwork and learning will benefit from knowing how the dynamics of these measures interrelate with one another in the context of evolving tasks and team behaviors.

In this representative design study, we ask whether elements of teamwork and taskwork can be neurodynamically separated? For this we draw from two EEG-derived neurodynamic frameworks, neurodynamic organizations and inter-brain coupling. Neurodynamic organizations are information-based abstractions, expressed in bits, of the structure of long-duration EEG amplitude levels. Neurodynamic information (*NI*, the variable of neurodynamic organization) is felt to continually accumulate as EEG amplitudes cycle through periods of persistent activation and deactivation in response to the activities and uncertainties of teamwork. The level of inter-brain EEG coupling during social interactions is estimated by wavelet transform coherence (*WCoh*) measures, based on the phase and amplitude of the EEG signals (Czeszumski et al., 2020).

### Inter-brain coupling

Simultaneous multi-brain recordings (hyperscanning) have often used the lower limits of neuroimaging technologies to document synchronized millisecond to seconds-long shifts in the EEG phase or amplitude (Lindenberger et al., 2009; Dumas et al., 2010; Hasson et al., 2012; Filho et al., 2016; Muller et al., 2021). Such inter-brain coordination during social interaction reflects temporal adjustments to brain network dynamics based on perceptions resulting from social interaction, or more recently from external modulation (Muller et al., 2021).

Outside the range of a few seconds, the ideas of interdependence among team members are mostly unexplored. There are indications that brain hyper-connections can occur independently in different people as intrinsic and extrinsic information become integrated over longer time scales (minutes or more; Hasson et al., 2008; Tranquillo and Stecker, 2016; Silva et al., 2019). These scrolling windows of cognition playing out well-practiced sequences of events are beginning to be described in individuals as temporal receptive windows (TRW), with elements of the Default Mode Network playing a role in integrating internal predictions of the future with the continuous updation of sensory information (Lerner et al., 2011; Yeshurun et al., 2021).

Examples include different individuals viewing the same movie segments or listening to the same narratives or music (Lerner et al., 2011; Clayton et al., 2020). Here the only dependency among people is the time the data stream starts, and again when different cognitive elements are sequentially activated internally by the unfolding sequence of sounds and events. Under these conditions the brainwaves being externally entrained could be coherent, but not necessarily coordinated (Burgess, 2013).

Such sequence entrainment/synchrony in real-world settings was shown to occur with submarine navigation teams (Stevens and Galloway, 2014, 2019, 2021b). The submarine navigation team consists of one group responsible for keeping the ship on course and they do so by checking and reporting the position every 3 min through a timed sequence of activities called Rounds. Meanwhile, another group is responsible for avoiding collisions and it does so by establishing possible collision targets using the course and direction of other ships. Prolonged periods (30–40 s) of time-ordered neurodynamic organization have repeatedly been observed with the submarine navigation group in parallel with time-ordered, recurring sequence of activities. Similar significant correlations among team members have been shown with healthcare teams as illustrated below. What has been lacking from these studies is information regarding the *IBC* among team members during these periods of neurodynamic organization. In fact, it is unknown whether prolonged periods of *IBC* are even produced during continuous simulation training.

### Neurodynamic organization and uncertainty

Uncertainty is a fundamental property of neural computation used by the brain to estimate the (perceived) state of the world. The brain draws from this uncertainty to access memories (the past) to imagine future possibilities and the actions needed to give the best outcomes. In this way, uncertainty serves as a trigger for adaptation (Knill and Pouget, 2004). While it is generally accepted that uncertainty should be avoided, it is also becoming apparent that uncertainty drives learning by triggering a switch from strategies exploiting past experiences to strategies exploring novel approaches (O’Rielly, 2013; Soltani and Izquierdo, 2019; Domenech et al., 2020; Gillon et al., 2021).

During teamwork, this exploratory uncertainty, and the pauses and hesitations it generates, are often early indicators of deteriorating performance (O’Riordan et al., 2011; Kaufman et al., 2015; Ott et al., 2018). Uncertainty is an intrinsic condition within healthcare that affects individual clinicians and teams during training and practice. Defined by Han et al.’s (2011) as, “a subjective perception of ignorance” uncertainty is messy and non-linear, and adds complexity to patient care that may result in patient harm since it is often a precursor state to error (Farnan et al., 2008). This fundamental perception of not knowing gives rise in conventional terms to doubts, hesitations, and lack of reliability in patient care (Han et al.’s, 2011). It likewise elicits a variety of behavioral and cognitive responses among clinicians (Lally and Cantillon, 2014; Nevalainen et al., 2014), and increases healthcare costs on a national and global scale (Dine et al., 2015). While hesitations represent a concern for the individual experiencing it, they also serve as an interruption to the team. Here, hesitation on the part of one team member interferes with work continuity and causes a resumption lag before the recommencement of the primary task. Most studies have shown that interruptions lead to a decline in performance (Zikerick et al., 2021).

Despite uncertainty’s ubiquitous presence, there has been little discussion about how to develop quantitative measures for detecting and modeling the dynamics of aggregated levels of uncertainty in teams. We have identified, and trained machines to recognize, neurodynamic correlates of uncertainty based on the pauses, hesitations, and verbalizations of teams (Stevens and Galloway, 2017, 2019, 2021a). These neurodynamic correlates are based on persistent information structures or neurodynamic organizations in EEG data streams.

EEG analyses are dictated by the physical units of amplitude, frequency, and phase of brain waveforms. Understandings of team behavior, however, are constructed around organizations, whether they be: production, personnel, distribution, or other variant structures (Mathieu et al., 2014). A useful transformation would be one that input physical units of EEG amplitude (μ-volts) and the output measures of organization (in bits), the rationale being that information-organization representation would better align with the organization-based measures of teamwork behaviors.

As detailed under “Methods” Section, the symbolic modeling generates a normative framework organization with 0, the information of a single symbol being the lower bound and the upper bound is the maximum information of a data stream containing a randomized set of the system symbols (i.e., 1.585 bits for a 3-symbol system, 3.17 for a 9-symbol system, etc.). The values between these bounds can be quantitatively compared across performances, or across brain regions or across the frequencies of the 1–40 Hz EEG spectrum (Stevens and Galloway, 2017).

Within this normative framework a measure termed Neurodynamic Information (*NI*), is generated which temporally bridges the gap between low level neural processes associated with everyday activities, and the hesitations and pauses associated with team member uncertainty (Stevens et al., 2018a; Stevens and Galloway, 2019, 2021b). The emerging picture from these studies is that as simulations and real-world events evolve, the accumulated *NI* of each individual becomes a measure of the frequency, magnitude, and duration of periods of uncertainty that have been experienced (Stevens et al., 2019).

In this way, a neurodynamic organization is a useful intermediate abstraction (Flack J. C., 2017) that contributes properties not always possessed by the amplitude or phase of brainwaves such as linking with the organization of team activities (Stevens and Galloway, 2017), or speech (Gorman et al., 2016), or the expertise of submarine or healthcare team proficiency (Stevens et al., 2018b; Stevens and Galloway, 2021b). In the spirit of representative design (Dhami et al., 2004; Nastase et al., 2020), and quantitative collectivity (Daniels et al., 2021) they also serve as the starting point for macro-scale to micro-scale cognitive deconstructions across temporal and spatial scales of brain dynamics where environmental properties are preserved.

## Methods

### Subjects and tasks

#### Medical flight teams

Five two-person medical flight teams performed a required pediatric patient simulation (acute bronchiolitis) within the interior of an emergency helicopter while wearing 19-sensor (Cognionics, Inc.) EEG headsets (112 min containing briefings and scenarios was recorded). All participants were experienced practitioners with 5 years or more in ICU-CCU settings who were participating in required training sessions. The sequence of events in each of the performances was an introduction to the task, an examination of the equipment and supplies available, a presentation of the patient’s history, and a short Q&A while on the tarmac. The team then entered the helicopter, assembled themselves with one person at the head of the patient (TM-1) and one at the side (TM-2). The team then managed the patient during the simulated trip to the hospital. The distribution of time in each segment varied for each team.

#### Medical student team

A second team with three 4th-year medical students managed a patient with a benzodiazepine overdose. This team performed simulations in a high-fidelity operating-room environment. The neurodynamics of this team and performance were previously studied in the context of speech to clarify the relationship between team communication and resolving uncertainty (Stevens et al., 2016).

### Ethics statement

Informed consent protocols were approved by the Biomedical IRB, San Diego, CA (Protocol EEG01), and the Order of Saint Francis Healthcare Institutional Review Board. Participating subjects consented (including images and speech for additional analysis) per approved applicable protocols. To maintain confidentiality, each subject was assigned a unique number known only to the investigators of the study, and subject identities were not shared. This design complies with DHHS: protected human subject 45 CFR 46; FDA: informed consent 21 CFR 50.

### Neurodynamic measures

#### EEG collection and pre-processing

Currently, the most common methods for removing artifacts in movement studies are methods based on independent component analysis (ICA) which transforms a set of vectors into a maximally independent set. EEG artifacts can be broadly divided into two classes: non-stereotyped artifacts due to multiple factors like the subject’s movements or external sources of interference, and stereotyped artifacts due, for example, to ocular eye movements, blinks, heartbeats (Onton et al., 2006). Artifacts from the second class are likely to be captured by some ICA components because they have a highly reproducible spatial distribution and temporal profiles. ICA decomposes the observed signals into independent components and after removing the unwanted components, the clean signal is reconstructed from the remaining independent components (Makeig et al., 1996). Artifacts from the first class are problematic for ICA because since their spatial distribution is extremely variable, they introduce a large number of unique scalp maps, leaving few ICs available for capturing brain sources. The data streams were therefore processed with a combination of ICA and artifact subspace reconstruction (ASR) which has several advantages including the automated removal of artifact components, its usability for online applications, and the ability to remove transient or large-amplitude artifacts that the ICA method struggles with (Kothe and Jung, 2014; Chang et al., 2018; Gorjan et al., 2022).

The 19 quick dry-electrode system sensors (CGX Cognionics Inc., San Diego) were designed with noise reduction into the hardware, with active electrodes, active shielding, and extremely low-noise electronics, and were fitted on each subject and then adjusted for good contact. When impedance was low (<10Ω) and the subject was ready, EEG data were continuously recorded with a sampling frequency of 500 samples/s from sensors positioned over the scalp according to the international 10/20 system, and all subject’s data streams were monitored throughout the session.

Although wireless systems enable mobility, accurate timing is difficult due to the inherent latency and jitter in wireless communications. The EEG data streams of team members, as well as audio and video recorders, were synchronized before and after each data acquisition with the Cognionics Trigger electronic time synch markers. The trigger system is created to accurately broadcast time markers with millisecond precision, resolving the issues of latency and jitter. The electronic time markers were also inserted during acquisition at task segment events like the end of the briefing, the beginning of a debriefing, or the start and finish of specific procedures like intubation. Because the timing accuracy is guaranteed in hardware, there is no need for software or algorithmic timing compensation (CGX Cognionics Inc., San Diego).

The time-synchronized EEG data were visually inspected to identify bad electrodes; these were not present in any of the study teams. Next, to remove linear trends and to obtain good quality ICA decompositions (Klug and Gramann, 2021), we high pass filtered the data at 1 Hz. To remove the 60 hz line noise, we applied the CleanLine EEGLAB (Mullen, 2012) plugin that adaptively estimated and removed sinusoidal noise coupling multi-tapering and a Thompson F-statistic. To aggressively remove transient and high amplitude medium to large artifacts that the ICA method struggles with, we applied ASR (EEGLAB Clean Rawdata) with the recommended cut-off parameter of *k* = 20, retaining approximately 20–40 percent of unmodified data. For stable decomposition, we applied the InfoMax independent component analysis (ICA) algorithm (runica) to detect and remove additional electrode drifts, eye movements, electromyographic and electrocardiographic interference (Delorme et al., 2007, 2011). All data sets were average referenced (Nunez and Srinivasan, 2006; Ludwig et al., 2009).

### Team neurodynamic modeling

The modeling goal was to develop a multi-modal, multi-level system that would provide neurodynamic measures from each team member at a 1 Hz resolution that could be quantitatively compared across sensor sites (i.e., the occipital lobe vs. the motor cortex) and the individual 1–40 Hz frequency bins from each person.

Detecting structure in data streams involves first deconstructing continuous EEG data into discrete symbols which requires choosing the number of partitions. Some EEG rhythms, like alpha waves (~10 Hz), show either enhancing or suppressive neurodynamic properties depending on whether they are in a high or low power state (Klimesch, 2012) and so at its simplest, EEG amplitudes of a team member could be assigned any three symbols such that the states are easy to visualize and understand. In our studies, activated states are assigned “3”, deactivated states are assigned “-1” and neutral states are assigned “1”. The result is a data stream of 3’s, 1’s, and -1’s.

**Figure 1:**
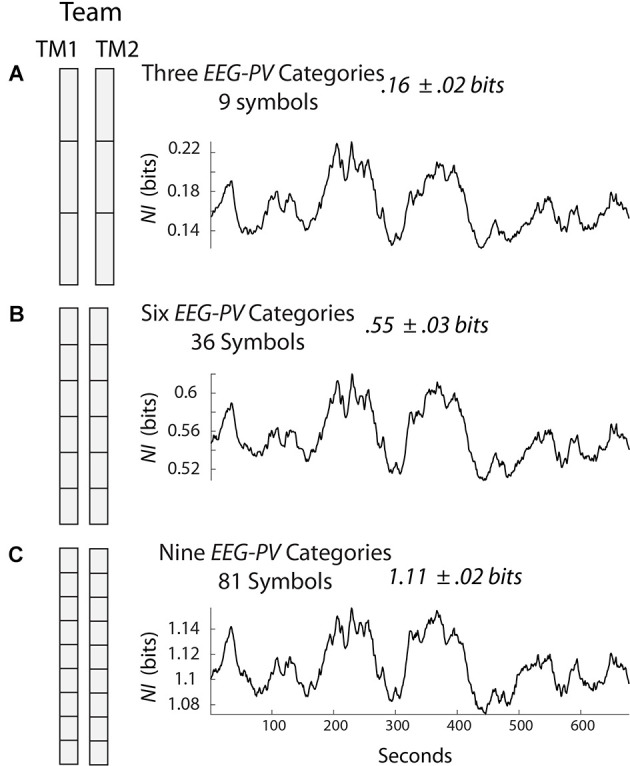
Symbolic modeling of neurodynamic data. The electroencephalography (EEG) was collected from two team members (TM1 and TM2) and each second the scalp averaged EEG amplitude values of each team member was separated into three **(A)**, six **(B)**, or nine **(C)** equal divisions. The *NI* was calculated for the three models using a 60 s moving window that was updated each second. NI, neurodynamic information.

Figure 1 shows a team of two persons where the EEG amplitudes were separated into three states each second (Figure 1A), six states (Figure 1B), or nine states (Figure 1C). Since there are two persons and three symbols in each person’s data stream in Figure 1A, the team data stream would have nine symbols. The temporal structure (not power) in this data stream can be estimated each second by measuring the mix (i.e., entropy) of the nine symbols in a 60 s segment that slides over the data and is updated each second. If only one of the nine symbols was expressed in this 60 s segment the entropy would be 0 bits; if there was an equal mix of the nine symbols, then the entropy would be 3.17 bits which is the maximum. So the fewer the symbols expressed in a window of 60 s the more organized the team was and the lower the entropy.

Neurodynamic Information (*NI*) is the information that remains when the experimental entropy values are subtracted from the maximum entropy for the number of unique system symbols. The *NI* profile for the team in Figure 1A is shown to the right and the average *NI* of the team’s performance was 0.16 bits. Similar calculations were made when the amplitude was separated into six or nine states (Figures 1B,C). Although the *NI* values increased with additional symbols in each group, the *NI* profiles were similar indicating that adding additional symbols had a negligible effect on the dynamical structure of the data; for most studies, the EEG data of each team member is separated into three categories.

Symbolically analyzing the structure of EEG amplitude creates a normative scale of EEG organizations ranging from 0 to the maximum *NI* of the number of symbols being used. A data stream with no organization would have an *NI* of 0. If the EEG were maximally organized the *NI* would be the maximum for the number of symbols in the system, i.e., 4.75 bits for a 27-symbol three-person team, 3.17 bits for a 9-symbol dyad, or 1.59 bits for a 3-symbol individual (i.e., high, average, low).

These mathematical limits have implications for creating quantitative performance measures. In other words, the *NI* of any two-person team performing a task where the EEG is separated into three PSD levels will have *NI* levels between 0 and 3.17 bits. The average value of 0.16 bits for the team in Figure 1A is one that can be quantitatively compared with other teams. If a team member’s average *NI* is calculated, this value can be quantitatively compared with that of other team members. Similarly, the neurodynamic organization of one brain region can be compared with that of another brain region and across the 1–40 Hz EEG frequency spectrum. The same reasoning applies if the team *NI* is compared during the simulation scenario vs. the debriefing, or during a critical healthcare event like intubation.

### Wavelet coherence

Methods for estimating inter-brain neural coordination are based on covariance in amplitude (Yun et al., 2012), or phase synchronization (Lindenberger et al., 2009). Wavelet transform coherence (*WCoh*) has also been used as an analytic tool, providing information on the level of coupling across brain regions of individuals or during social interactions (Czeszumski et al., 2020). Wavelet coherence is useful for analyzing nonstationary signals and considers both the phase and amplitude of the signals.

For deriving wavelet coherence coefficients the EEG data streams were down sampled to 1 Hz to parallel the dynamics of the EEG power spectrum density (PSD) estimates used for determining neurodynamic information.

The *IBC* between the two team members was made at the sensor level using the Matlab^®^ function *wcoherence.m*. This function returns the magnitude-squared wavelet coherence, which is a measure of the correlation between signals x and y in the time-frequency plane. A similar analysis was performed for data streams that had been randomized three times and these values were subtracted from the performance values.

Wavelet coherence is most useful for measuring how similar the power and phase are at each frequency of the two signals, and are robust for non-directed functional connectivity studies like ours (Bastos and Schoffelen, 2016). Other source information measures of connectivity like the Source Information Flow Toolbox (SIFT; Delorme et al., 2011) are more appropriate if directional, causal connectivity analyses are being considered as they are less sensitive to volume conduction effects.

**Figure 2:**
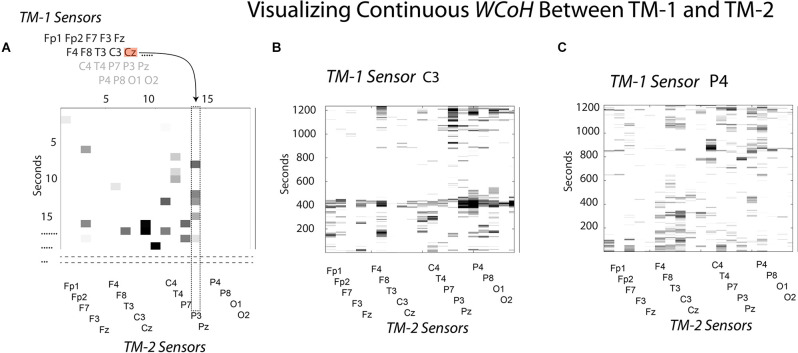
This figure illustrates how *WCoh* and *MI* were calculated across sensors for a dyad. **(A)** The neurodynamic measures at the sensors of TM-1 were sequentially modeled with those of TM-2 at each second to calculate the *IBC*; refer to the text for details. **(B)** The *WCoh* pattern after the neurodynamics of TM1’s C3 sensor were passed over the 19-sensors of TM-2. **(C)** The *WCoh* pattern after the neurodynamics of TM1’s P4 sensor were passed over the 19-sensors of TM-2.

To visualize the sensor × time *WCoh* dynamics, the EEG data stream of the first sensor (Fp1) of TM-1 were sequentially used to make wavelet coherence coefficients in combination for each of the remaining 18 sensors of TM-1, resulting in a 19-sensor wavelet coefficient map of the performance for the Fp1 sensor. This would be repeated with the second sensor of TM-1 to create another 19-sensor performance map, and so on until the 19 × 19 × time maps were completed. The diagram in Figure 2A illustrates the point in the modeling where the Cz sensor of TM-1 is being used in conjunction with the P3 sensor of TM-2, the previous 13 sensors of this map having been completed.

Two of the 19 *WCoh* sensor × performance maps are shown in Figures 2B,C, the first where the EEG data of the C3 sensor of TM-1 had been passed over the 19 sensors of TM-2, and the second map after the P4 sensor of TM-1 had been modeled. These two maps were selected to show the variability across *WCoh* maps. The first illustrates strong coherence around 400 s where the activity in the C3 sensor of TM-1 shows coherence with most of the sensors of TM-2 (i.e., a global form of coherence). This activity was missing when the P4 EEG of TM-1 was used instead of the C3 sensor. While the different *WCoh* maps show large temporal variability, the peaks within each map were sparse and discrete. These findings are representative of the remaining 17 *WCoh* maps which are not shown.

The *MI-*determined couplings across brain regions were visualized as described above for *WCoh*.

## Results

### Scalp-wide averages of *NI*, *MI*, and *WCoh*

**Figure 3:**
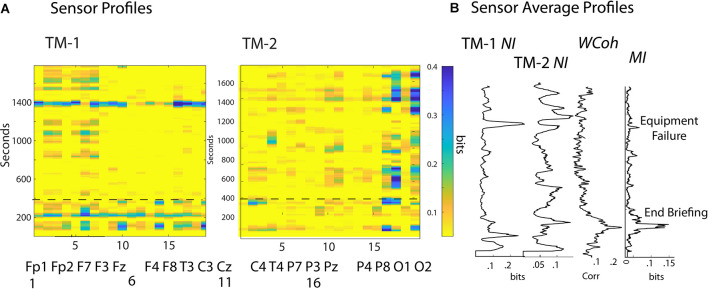
**(A)** The frequency-averaged *NI* values are plotted for each sensor every second for TM-1 at the Head position, and TM-2 at the Side position. The dotted line separates the Briefing and Scenario portions of the performance. **(B)** Profiles are shown for the sensor-averaged values of *NI* of TM-1 and TM-2; and the *WCoh* and *MI* of the dyad. The labels beside the *MI* plot indicate when the Briefing ended and when the ventilator failed to initialize (~1,400 s). There was a positive *MI*-*WCoh* correlation in **(B)** (*r* = 0.68, *p* < 0.05).

The scalp-wide *NI*, *MI*, and *WCoh* levels were calculated using EEG-frequency and sensor averaged values for seven healthcare dyads. The average *NI* level was 0.09 bits, and the *MI* was 0.007 bits, or 7.7% of the dyad’s average *NI* levels. The *NI* and *MI* values were both similar to previously published averages for a mix of 49 healthcare, military, and undergraduate dyads (Stevens et al., 2018a). *WCoh* levels are measured in terms of percent coherence and cannot be quantitatively compared with *NI* or *MI* (Figure 3).

The *NI* correlations between the members of each dyad were variable, averaging *r* = 0 0.37, *p* = 0.02 with a range of *r* = -0.02–0.54. There were no correlations between the *NI* of the team members and the *WCoh* (*r* = 0.08), or the *MI* (*r = -0.06)* of their dyads. There was a weak but non-significant (*r* = 0.27, *p* = 0.26) positive correlation between *MI* and *WCoh* levels.

### Temporal and spatial profiles of *NI*, *MI*, and *WCoh*—example 1

#### Overall dynamics

The analyses first explored the temporal (across the time of the performance) and spatial (sensors across the scalp) neurodynamics of *NI*, *MI*, and *WCoh* (Figure 3). The measure dynamics are displayed each second as frequency-averaged values for each of the 19 sensors in (Figure 3A) or as the sensor-averaged profiles of the performance (Figure 3B).

The surface maps sequence the sensors from the frontal scalp positions on the left of the maps, towards the rear of the scalp on the right. The frontal region sensors were those numbered 1–7, the central region sensors were in columns 8–12, the parietal region sensors were in columns 13–17, and the occipital region sensors were in columns 18 and 19.

The *NI* activity of both TM-1 and TM-2 was sparsely distributed at both the temporal and spatial levels with most peaks discrete and lasting 20–40 s. At any time, the elevated *NI* could be limited to a few sensors or be more globally distributed. During much of the Scenario, the elevated *NI* of TM-1 was in the frontal region (sensors 2–3, 5–7), while elevated *NI* of TM-2 was mainly in the parietal (sensors 16–17) and the occipital (O2) regions.

The sensor-averaged profiles (Figure 3B) of *MI* and *WCoh* indicate that much of this activity was during the Briefing when the instructor presented the patient’s history and flight direction plan. There was a parallel, more discrete *MI* peak that aligned with the center of the *WCoh* peak. These peaks coincided with a period of low team *NI* levels when both team members were silent, still, and attentive.

#### Briefing dynamics

**Figure 4:**
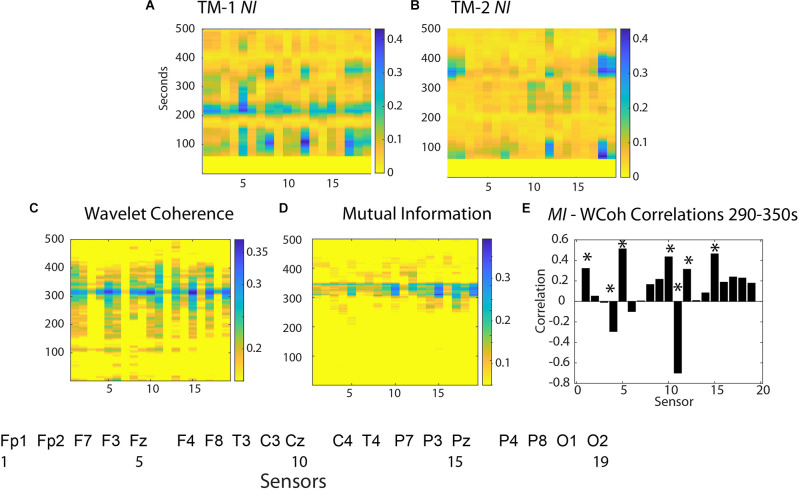
Neurodynamics of the Briefing Segment (1–500 s). The *NI* values are plotted at each sensor for **(A)** TM-1 and **(B)** TM-2. The bottom row shows surface plots for **(C)** *WCoh* and **(D)** *MI* values. **(E)** This figure plots the *WCoh-MI* correlation at each sensor for 1,290–1,350 s; correlation *p*-values < 0.01 are indicated by asterisks (*).

An expansion of the 1–500 s Briefing segment (Figures 4A,B) shows the majority of the *NI* of TM-1 occurred at sensor positions Fz, T4, P8, and O2 (# 5, 12, 17, 19), while those of TM-2 *NI* were simultaneously elevated in the C4 and O1 sensors.

The *WCoh* activity was broadly distributed from ~150 s until ~450 s with a multi-sensor prominent peak at ~325 s when the *NI* levels of TM-1 and TM-2 were low (<0.05 bits). The elevated *WCoh* activity was globally distributed across the scalp being present in all sensors except at F7 and F8 (sensors 3, 7) in the frontal region, and P3, P4, T4, and O1 (sensors 14, 16, 12, and 18) in the parietal/occipital regions (Figure 4C).

The *MI* was more restricted over a 45 s period with the highest levels in the Fp1, F3, Fz, Cz, C4, T4, and Pz sensors (1, 4, 5, 10, 11, and 15; Figure 4D). The correlation between scalp-averaged *MI* and *WCoh* was *r =* 0.68 for the performance, and a channel-by-channel analysis showed that the highest (positive) correlations were at sensors Fz (5), Cz (10), Pz (15), and (negative) at C4 (11) (Figure 4E).

These results suggest that when present, the *WCoh* and *MI* levels were elevated outside the times of elevated *NI* and that they were more globally expressed than the peaks of *NI* in the Briefing.

#### Equipment failure

**Figure 5:**
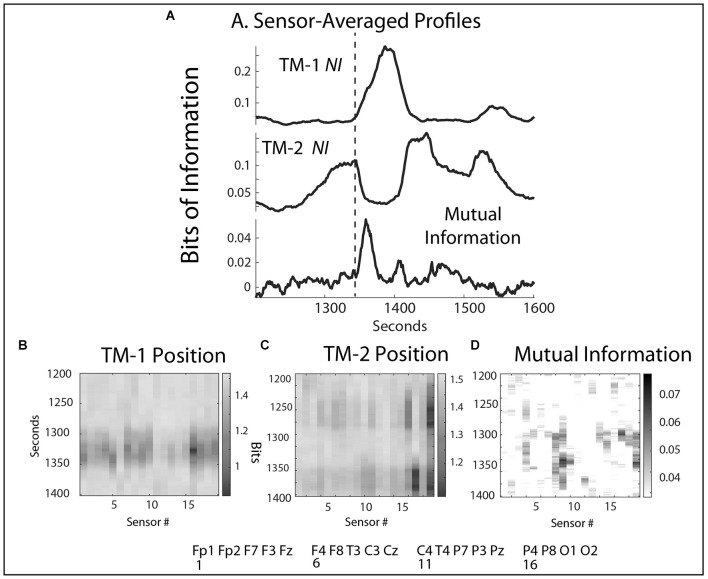
**(A)** Sensor-averaged profiles of the *NI* from TM-1 and TM-2, and the *MI* during the same episode. The dotted line indicates the start of the *MI* elevation. **(B,C)** The *NI* sensor profiles during the 1,300–1,500 s interval. **(D)** The average *MI* sensor profile over the same interval.

The rhythm of the team was perturbed between 1,300–1,500 s when the ventilator machine failed to initialize properly for the size and weight of the infant. During this period, the *NI* levels of both TM-1 and TM-2 increased, although not in parallel (*r* = -0.49, *p* = 0.05). The elevated levels of *NI* with peaks >0.4 bits indicated high levels of uncertainty, confirmed by statements like. “How come I can’t… and It’s not coming up like usual for non-invasive.” There was also a small peak of *MI* in the profile in Figure 5A.

The sequence of events during this period began at 1,280 s with TM-2 focusing on arranging the breathing hoses prior to attaching them to the ventilator and the baby. These activities were associated with elevated *NI* at the P4 and O2 sensors (16, 19) (Figures 5A,C). When TM-2 changed tasks and began assisting TM-1, her *NI* activity rapidly decreased, and at the time when both team members’ *NI* was low (~1,340 s), the *MI* rose while they co-entered settings into the ventilator (Figures 5A,B,D). When the team realized the machine was not initializing properly, the *NI* of TM-1 elevated in the frontal regions and decreased in the sensorimotor regions for ~45 s as she worked unsuccessfully to reset the machine. Around 1,370 s, the *NI* activity in the Pz and O2 sensors of TM-2 rose as she assumed control in adjusting the settings. At 1,420 s the instructors intervened to help reset the machine and by 1,500 s the Scenario continued.

### Estimating the frequency, magnitude, and duration of *NI* and *MI* peaks

**Figure 6:**
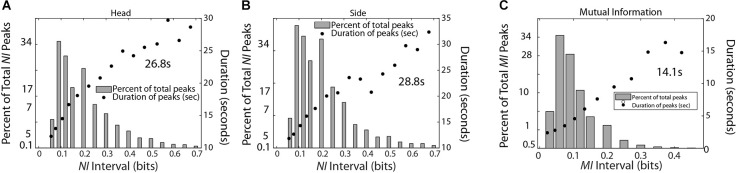
This figure shows the relationship between the magnitude and duration of the *NI* peaks for TM-1 **(A)** and TM-2 **(B)**, and the *MI* of the dyad **(C)**. The mean duration was calculated for each panel from the top five data points for **(A,B)** and the top four data points for **(C)**.

Scalp-averaged *NI* levels, representing periods of uncertainty vary based on their frequency, magnitudes, and durations of neurodynamic organization, while for *MI* and *WCoh*, these characteristics would apply to periods of *IBC*. The frequency and magnitude of *NI* peaks can be estimated by peak-finding routines that identify peaks based on the magnitude and the relationships with their neighbors. One function is Matlab^®^ *findpeaks.m* which identifies a peak as being a data sample that is larger than its neighbors and has a specified prominence (magnitude). In addition, the function calculates the extent (duration) of the peak at half prominence. For the data in Figure 6, the data was considered a peak if it was at least 0.05 bits larger than neighboring peaks. Subsequently, the peak measures were selected based on them being within the intervals ranging from 0.005 to 0.7 bits for Figures 6A,B or 0.005 to 0.4 for Figure 6C.

The data stream for TM-1 was a concatenation of the 1–40 Hz frequency bins for each sensor for both team members (total epochs = 1,288,770). The occurrence of *NI* peaks was 0.074 for both TM-1 and TM-2 while the occurrence of *MI* peaks was 0.016 for the dyad. The *MI* was chosen for this comparison as the peaks were more discrete than those of *WCoh*. The peak durations for the *NI* of TM-1 and TM-2 were 26.8 s and 28.8 s, respectively while those of *MI* were 14.5 s.

### Medical flight team neurodynamics—example 2

**Figure 7:**
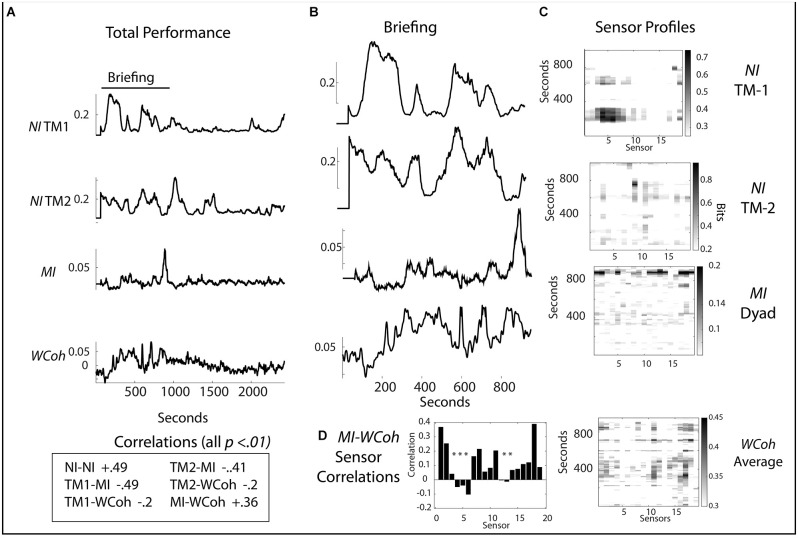
**(A)** Scalp averaged profiles of the *NI* and *MI* of TM-1 and TM-2 are shown for the entire performance. **(B)** Scalp-averaged profiles for the Briefing segment of the performance. **(C)** Sensor profiles are shown for the *NI* of TM-1 and TM-2, as well as for the *MI* and *WCoh* during the Briefing segment. **(D)** Correlation between *MI* and *WCoh* for the Briefing Segment; the asterisks indicate *p* < 0.01.

The neurodynamic profiles of a second medical flight team are shown in Figure 7. In this performance the team members’ *NI*-*NI* correlation was (*r* = 0.49, *p* < 0.01) and there were negative correlations between *MI* and the team member’s *NI* (*r* = -0.49, *p* < 0.01 and *r* = -0.41, *p* < 0.01) for TM1 and TM2 and between *WCoh* and *NI* for the team members (*r* = -0.20, *p* < 0.01 and *r* = -0.30, *p* < 0.01). The correlation between the team’s *MI* and *WCoh* values was *r* = 0.36, *p* < 0.01.

Like the first team performance, there was greater *NI*, *MI*, and *WCoh* activity in the Briefing for this team. The first *NI* segment for TM-1 (120–318 s) occurred when the dyad was developing its management plan for the infant. Then TM-1 entered the helicopter (474–800 s) to initialize the onboard medical equipment (Figures 7A,B). The major *NI* peak for TM-2 occurred between 955 and 1,160 s inside the helicopter after the Briefing and when patient management began. There were also minor peaks ~732 and 793 s (Figures 7B,C) when TM-2 was watching TM-1 initialize the machines; this *NI* activity was centrally (C3 and C4 sensors) located (Figure 7C).

The major *MI* profile between 830 and 952 s occurred while the *NI* of both team members was low. This peak was more uniform than the parallel peak of *WCoh*. The broad region of *WCoh* activity from ~200 to 600 s was visible in the *MI* profile but at a low rate. The highest *WCoh* levels were in sensors C4 (11) and P4 (16). These were also present in the major *MI* profile along with others in the frontal [Fp1 (1), Fp2 (2), F4 (6) T4 (12), and parietal regions P3 (13), P7 (14), P8 (17)]. The *WCoh—MI* correlation was greatest at the Fp1 (1) and O1 (18) sensors.

### Example 3 - three-person medical student team

The above experienced team studies showed that persistent periods of elevated *MI* and *WCoh* were present when elevated periods of *NI* were low. These persistent *IBCs* were often at sensor locations different from those contributing to elevated *NI* and were of smaller magnitude and shorter duration. There were also correlations between the mutual information and the wavelet coherence measures that differed with the sensor location.

The final example modeled the neurodynamics of three fourth-year medical students (designated Red, Green, and Blue) managing a patient with a benzodiazepine overdose. No team roles were assigned to the students, and individual activities were informally decided as the case evolved. For instance, the Blue team member calculated doses, while the Red team member led the intubation procedure with Green assisting.

**Figure 8:**
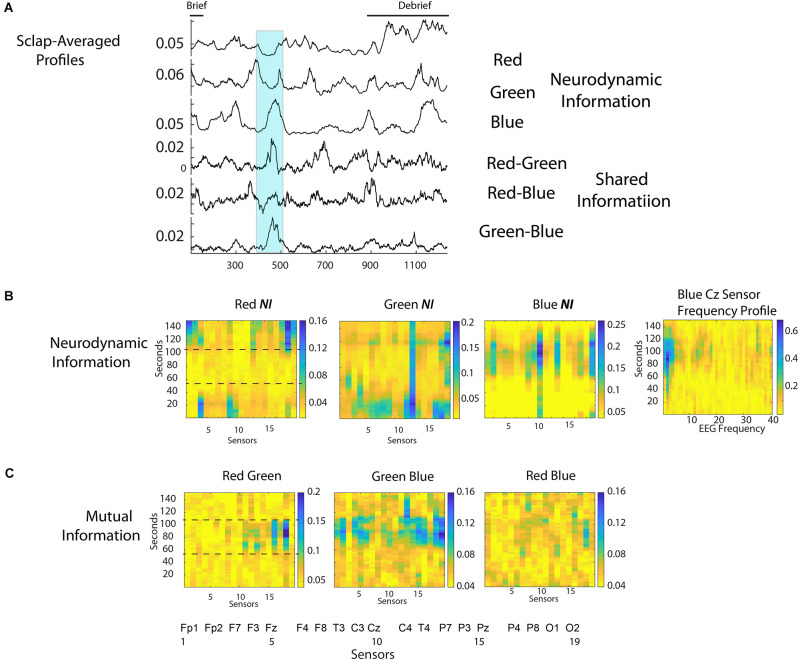
**(A)** The scalp-averaged *NI* levels for the Red, Green, and Blue team members, and the *MI* for the Red-Green, Red-Blue, and Green-Blue dyads respectively. **(B)** The *NI* levels for the Red, Green, and Blue team members during the 150 s interval when the patient was intubated (350 s to 500 s of the performance). The figure to the right of the first row is the 1–40 Hz frequency profile for the Cz sensor of Blue. **(C)** The *MI* for the Red-Green, Green-Blue, and Red-Blue dyads during the 151 s intubation segment.

The scalp-averaged *NI* values for Red, Green, and Blue were 0.039 bits, 0.052 bits, and 0.063 bits respectively, and the dyadic *MI* levels were: Red-Green, 0.054 bits; Red Blue, 0.055 bits, Green-Blue, 0.065 bits. Mutual information is used in this example as a measure of *IBC* as it provides more discrete peak profiles than *WCoh*. The scalp-averaged *NI* and *MI* profiles are shown in Figure 8A. There was a short Briefing at the beginning, and a more extensive Debriefing at the end of the simulation than with the medical flight teams.

The most prominent *MI* peak coincided with the decision to intubate the patient and continued during the two attempts that followed (the second one was successful). This segment is shown by the highlighted region from 375 to 525 s. This 151 s segment was accompanied by elevations in *NI* and *MI* for different team members prompting the more detailed analysis in Figure 8B. During this 151 s period, the team was relatively quiet with Red speaking 28 s and Green and Blue speaking 40 s and 13 s, respectively.

The *NI* levels were greatest for Blue and Green when the decision to intubate was being made, and when the second attempt at the intubation procedure occurred. For Blue, there was a large peak of *NI* at the Cz sensor when the first intubation was unsuccessful and when the second attempt began. A further analysis at the frequency level indicated most of the activity was around 2–4 Hz. A similar peak of theta band activity around the Cz sensor has been associated with an interruption-based deterioration of task performance (Zikerick et al., 2021).

The lower dotted line in the first panel of Figures 8B,C indicates the point when the decision to intubate had been made, while the upper dotted line indicates when the second, and successful intubation procedure started. Most of the *IBC* occurred between these two lines. In other words, the greatest *IBC* occurred while the team watched/participated in the first, and unsuccessful intubation.

**Figure 9:**
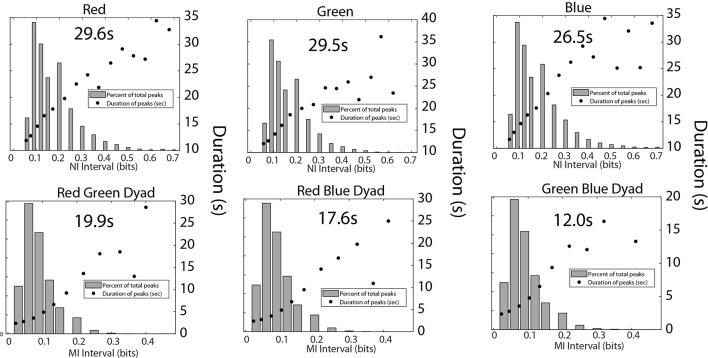
This figure shows the relationship between the magnitude and duration of the *NI* peaks for the Red, Green, and Blue team members (top), and for the *MI* peaks for the Red-Green, Red-Blue, and Green-Blue dyads (bottom). The mean duration was calculated for each panel from the top five data points for the top row figures, and the top four data points for the bottom row figures.

A final set of analyses were performed with the medical student team to estimate the frequency, magnitude, and duration of *NI* and *MI* of the team members (Figure 9). Consistent with the medical flight team findings in Figure 6, the average duration of the *NI* peaks was 28.3 ± 3.8 s while those of the *MI* was 16.0 ± 5.1 s (*t* = 8.02, *p* < 0.001).

## Discussion

We have compared the neurodynamics of healthcare teams across time and brain regions during autonomous (individuals resolving uncertainty) and collaborative (wavelet coherence and mutual information) segments of activity to determine whether their dynamics were independent, interdependent, or perhaps mutually exclusive. Uncertainty, as measured by *NI*, is often a persistent state (>15 s; Stevens and Galloway, 2014, 2017, 2019), is multifractal (Likens et al., 2014), and can be decomposed into periods of shorter duration (Figures 6, 9). The practical benefits from obtaining evidence higher up the temporal hierarchy of cognition, and closer to observable behaviors is that the system may be amenable to change through interventions.

While both *NI*-related measures of uncertainty and inter-brain coherence have histories of operating at small time frames such as milliseconds-seconds, and while periods of elevated *NI* have been shown to persist over time frames of seconds to minutes, it was unclear how common persistent *IBC* states were during real-world task performance.

Evidence that persistent *IBC* states may exist has come from dissecting the structures of a neurodynamic organization during the continuously evolving tasks (Stevens et al., 2018b). These studies showed that continuous quantitative estimates of team *NI* could be deconstructed into those of the individual team members, and across 49 dyads performing in different teaming domains, the sum of the team member *NI* accounted for ~90% of that of the team *NI*. There was always a residual amount (3%–15%) of information that was shared among the team members (Stevens et al., 2018a). These periods of neurodynamic mutual information were often distributed throughout the task and briefing/debriefing segments but were poorly correlated with changes in the *NI* of team members or the speech patterns of the teams (Stevens and Galloway, 2015; Stevens et al., 2017). In other words, it was not clear whether *MI* reflected inter-brain coupling in the sense of that modeled by other inter-brain measures like coherence (Bastos and Schoffelen, 2016), or whether it represented other forms of information sharing (or creation) activity among team members.

Persistent states of *WCoh* were observed in all teams in this study. The across subject scalp-averaged *MI* and *WCoh* levels of the seven performances were not correlated (*r* = 0.27. *p* = 0.26), but became so (i.e., Figures 3, 7) when individual teams were studied. When measured within subjects at the EEG sensor level (Figures 4, 7), correlations were large and significant. The *WCoh*-*MI* sensor-level correlations showed that although there was often a close concordance between *MI* and *WCoh* activities, they were not identical, showing both positive and negative sensor-level differences. This diversity may be from *WCoh* measures being derived from both the power and phase and *MI* being power-derived.

Nevertheless, examples of persistent (>15 s) *IBC*, both *WCoh* and *MI* derived, were found in all teams studied, and most frequently present during the briefings and debriefings where the simulation was being framed or discussed, respectively. Briefings are a critical part of simulation training as it is when the instructor gives the patient history (Petranek, 1994; Fanning and Gaba, 2007). During these times, the dyads were generally still and mostly silent. These segments may represent organizations that occur when the rhythm of the team members has been captured or entrained by task elements and/or the actions of other members, like the instructor (Adrian and Matthews, 1934; Galambos et al., 1981). As an extension, they also resemble periods of complex collective cognition while groups view emotionally-rich movie scenes (Hasson et al., 2004; Domachowski et al., 2012).

At any moment, increased *IBC*, as well as *NI* could be found in a single or across multiple sensor channels. Observationally, the number of sensors involved was related to the level of the measure. From a cognitive perspective, this would be consistent with the distributed nature of uncertainty (Grupe and Nitschke, 2013) and would represent an expansion from a local to a more global search (Lewis et al., 2019). From a network perspective, a larger and more connected network allows a perturbation to propagate across the network and results in more system amplification. At a critical point (critical amplification) a perturbation can grow to encompass a significant faction of system resources (Daniels et al., 2017). The neurodynamic magnitude and duration curves shown in Figures 6, 9, suggest that the critical amplifications for *NI* and *MI* may be reached ~30 s for *NI* and half that for *MI* (~15 s). These estimates may provide the durations within which to work for training interventions.

The most notable differences during periods of *IBC* and *NI* were their temporal dynamics. Previously we showed in a variety of teaming situations that *NI* levels elevate during periods of uncertainty, similar to those experienced by the equipment failure in Figure 5 and the intubation attempts in Figure 8. Han et al.’s (2011) emphasis on uncertainty being a “subjective perception” highlights the singular nature of the state.

When measured at the aggregated scalp level there were no positive correlations between the *IBC* and *NI* suggesting that the factors elevating and resolving uncertainty are singular processes and that the involvement of increased *IBC* is minimal.

The closest example of simultaneous *IBC* and *NI* dynamics is shown in Figure 5 during the over 3-min segment while the team tried to resolve the equipment failure. Even here, the elevated *IBC* occurred during a short gap when the *NI* decreased for both TM-1 and TM-2.

Elevated levels of *MI* were near, but not coincident with periods of *NI* (Stevens and Galloway, 2015). A similar relationship was seen in this study where the temporal difference of both *WCoh* and *MI* varied for tens of seconds away from *NI* peaks (Figures 4, 5) to a minute or more away (Figure 7). The significance of these temporal associations is unknown.

Neurodynamic information in the context of uncertainty exists, at least partially, as a conscious (i.e., to be verbalized) and observable aggregate behavior with hesitations and pauses. The possibility exists that the lack of success (to date) in linking* MI* with behaviors may mean that *MI* and *(WCoh)* are more unconscious intermediate representations between the micro and macro layers of teamwork, and are those that influence subsequent aggregate behaviors, but do not directly participate in them (Flack J., 2017).

Nevertheless, these studies show that the persistent expressions of *NI* and *MI* were not simultaneous, suggesting that it may be difficult for team members to maintain inter-brain coherence while simultaneously reducing their individual uncertainties (and vice versa).

A mechanism behind these observations might be resource allocation. While an attractive candidate for such a resource would be working memory (Huynh Cong and Kerzel, 2021), the temporal timeframe of working memory is generally much shorter than the time frames being modeled here. The models being generated in this study, however, are amenable to being studied over shorter time frames.

A second possibility is that individual and shared information represent phases of the collective decision-making process. From millisecond neuronal decision-making to crowd sourcing, collective decision-making shows bi-phasic properties (Daniels et al., 2017) with the accumulation of evidence by individuals preceding a more rapid group consensus-building phase.

During teamwork, the dynamics of individual information would represent the accumulation of evidence by each team member. In the second phase, the accumulated evidence would be integrated across team members into a decision through a more-rapid information sharing process. The attractiveness of this model is: (1) that periods of increased individual information would be temporally more prolonged than those of the shared information, and (2) much of the shared information would occur outside the times of the maximum individual neurodynamic organization, trends consistent with *NI* and *MI* dynamics.

### Limitations and future studies

This is an exploratory study subject to the challenges and limitations of teams, tasks, sample sizes, and the large temporal scales over which the performances were collected. Nevertheless it provides evidence that teamwork and taskwork are not always interdependent and may be mutually exclusive when measured at scales close to observable functional outputs. It also suggests future directions. For instance, network graphs of the segments before, during, and after perturbations will provide quantitative estimates of the shifting network structures of both *NI* and *IBC*, and refined views of possible interdependencies. For *WCoh*, and particularly *MI*, similar analyses can be performed within smaller brackets of duration (and magnitude) to better understand the finer temporal dynamics leading to the critical amplifications characterizing uncertainty.

In this article, few attempts have been made to provide interpretations for the brain region spatial and connectivity expressions of *NI*, *MI*, and *WCoh* in the context of the task events. Our previous experiences suggest that the use of machine learning tools (Stevens and Galloway, 2019) might be a worthwhile approach for determining the neurodynamic relationships of *IBC* and *NI* across sensors and frequencies. The discrete nature of *NI* and *MI* peaks at a 1 s resolution would facilitate the search for these peaks/motifs.

## Data Availability Statement

The datasets presented in this article are not readily available because the data will be available to the extent that it is allowed by the Institutional Review Board agreements. Requests to access the datasets should be directed to info@teamneurodynamics.com.

## Ethics Statement

The studies involving human participants were reviewed and approved by Biomed IRB, San Diego CA, and the Order of Saint Francis Hospital Institutional Review Board. The patients/participants provided their written informed consent to participate in this study. A comprehensive ethics statement is included in “Methods” Section.

## Author Contributions

RS and TG co-designed the study and wrote the article. Both authors contributed to the article and approved the submitted version.

## Conflict of Interest

RS received his PhD in Molecular Genetics from Harvard University and is Professor (Emeritus), UCLA School of Medicine and a member of the UCLA Brain Research Institute. RS was also employed by The Learning Chameleon, Inc., a for profit educational technology corporation. His recent research had focused on using EEG-derived measures to investigate team neurodynamics in complex, real-world training settings. TG was employed by, and was the director of cognitive electrophysiology research, and Facility Security Officer for The Learning Chameleon laboratory. She received her CPFDA, EFDA and CDA from Oregon Health and Sciences University in 1995 later specializing in several areas of process analysis.

## Publisher’s Note

All claims expressed in this article are solely those of the authors and do not necessarily represent those of their affiliated organizations, or those of the publisher, the editors and the reviewers. Any product that may be evaluated in this article, or claim that may be made by its manufacturer, is not guaranteed or endorsed by the publisher.
